# Peripheral Serotonin 1B Receptor Transcription Predicts the Effect of Acute Tryptophan Depletion on Risky Decision-Making

**DOI:** 10.1093/ijnp/pyw075

**Published:** 2016-09-16

**Authors:** Paul Faulkner, Federico Mancinelli, Patricia L Lockwood, Mar Matarin, Raymond J Dolan, Nick W Wood, Peter Dayan, Jonathan P Roiser

**Affiliations:** 1 Institute of Cognitive Neuroscience, University College London, London, United Kingdom (Drs Faulkner and Roiser); Psychiatry and Biobehavioral Sciences, Semel Institute, University of California, Los Angeles, California (Dr Faulkner);; 2 Gatsby Computational Neuroscience Unit (Mr Mancinelli and Dr Dayan), and CoMPLEX Centre for Mathematics, Physics and Engineering in the Life Sciences and Experimental Biology (Mr Mancinelli), University College London, London, United Kingdom; Experimental Psychology, University of Oxford, Oxford, United Kingdom (Dr Lockwood); Clinical and Experimental Epilepsy, Institute of Neurology (Dr Matarin), and Wellcome Trust Centre for Neuroimaging (Dr Dolan), University College London, London, United Kingdom; Molecular Neuroscience, UCL Institute of Neurology, Queen Square, London, United Kingdom (Dr Wood).

**Keywords:** serotonin, decision-making, acute tryptophan depletion, 5-HT1B, risk

## Abstract

**Background:**

The effects of acute tryptophan depletion on human decision-making suggest that serotonin modulates the processing of rewards and punishments. However, few studies have assessed which of the many types of serotonin receptors are responsible.

**Methods:**

Using a within-subject, double-blind, sham-controlled design in 26 subjects, we examined whether individual differences in serotonin system gene transcription, measured in peripheral blood, predicted the effect of acute tryptophan depletion on decision-making. Participants performed a task in which they chose between successive pairs of fixed, lower-stakes (control) and variable, higher-stakes (experimental) gambles, each involving wins or losses. In 21 participants, mRNA from 9 serotonin system genes was measured in whole blood prior to acute tryptophan depletion: 5-HT1B, 5-HT1F, 5-HT2A, 5-HT2B, 5-HT3A, 5-HT3E, 5-HT7 (serotonin receptors), 5-HTT (the serotonin transporter), and tryptophan hydroxylase 1.

**Results:**

Acute tryptophan depletion did not significantly influence participants’ sensitivity to probability, wins, or losses, although there was a trend for a lower tendency to choose experimental gambles overall following depletion. Significant positive correlations, which survived correction for multiple comparisons, were detected between baseline 5-HT1B mRNA levels and acute tryptophan depletion-induced increases in both the overall tendency to choose the experimental gamble and sensitivity to wins. No significant relationship was observed with any other peripheral serotonin system markers. Computational analyses of decision-making data provided results consistent with these findings.

**Conclusions:**

These results suggest that the 5-HT1B receptor may modulate the effects of acute tryptophan depletion on risky decision-making. Peripheral levels of serotonin markers may predict response to treatments that act upon the serotonin system, such as selective serotonin reuptake inhibitors.

**Significance Statement**

In this manuscript, we show that the effects of depleting central serotonin levels, via acute tryptophan depletion, on risky decision-making are moderated by peripheral levels of serotonin (5-HT)1B mRNA. The current results extend those of a much-cited study (Rogers et al., 2003) - which show acute tryptophan depletion to reduce sensitivity to wins on a risky decision-making task - by revealing that the ability of this treatment to affect such reward processing is moderated by peripheral 5-HT1B transcription. The results of this study highlight the importance of considering individual differences when examining responses to this treatment, which is utilized by many research groups. Further, they suggest that examining peripheral levels of serotonin markers may help to predict response to treatments that act upon this neurotransmitter system, such as selective serotonin reuptake inhibitors.

## Introduction

The serotonin (5-HT) system has long been implicated in decision-making (Soubrie et al., 1986; Deakin and Graeff, 1991), yet its precise role in reward and punishment processing remains unclear (Dayan and Huys, 2009; Boureau and Dayan, 2011). Several studies have attempted to characterize this relationship by manipulating 5-HT levels via the dietary technique of acute tryptophan depletion (ATD; see Faulkner and Deakin, 2014 for a review). The results of such studies are mixed, but a common finding is that ATD can influence the processing of punishments on reaction time tasks (e.g., Cools et al., 2008; Crockett et al., 2012; Robinson et al., 2012). For example, Crockett et al. (2009) showed that ATD abolished punishment-induced behavioral inhibition on a go/no-go task; and Geurts et al. (2013) extended these results, reporting that ATD led to decreased punishment-induced instrumental inhibition while reversing the inhibitory effect of aversive (but not appetitive) Pavlovian stimuli on instrumental responding (replicated by Hebart and Glascher, 2014). Studies of the effects of ATD on reward processing are also mixed. While Cools et al. (2005) found that ATD attenuated reinforcement-related speeding on a cued-reinforcement reaction time task (partially confirmed by Roiser et al., 2006), den Ouden et al. (2014) reported that ATD decreased behavioral inhibition to a similar extent for both rewarding and punishing stimuli on a modified cued-reinforcement reaction time task, though only in a high-feedback probability condition.

Studies that have examined the effect of ATD on probabilistic choice tasks have also produced mixed results. Rogers et al. (2003) administered a gambling task that assessed participants’ choices as a function of (1) probability, (2) the magnitude of expected gains, and (3) the magnitude of expected losses. The authors found that ATD altered participants’ decision-making by attenuating their discrimination between large and small wins, while the processing of losses and probability was unaffected. Seymour et al. (2012) observed a result weakly consistent with this using a probabilistic 4-armed bandit task: ATD altered the exchange rate by which rewards and punishments were compared, with this alteration being driven by a decrease in the subjective value of rewards. However, Rogers et al. (1999b) observed an ATD-induced reduction in high probability choices on the Cambridge Gamble Task, while Talbot et al. (2006) found the opposite result. The discordant results between these latter 2 studies suggest a need to examine the potential role of individual differences in 5-HT function in moderating the effects of ATD.

Very few studies have attempted to delineate the roles of specific receptors in the effects of 5-HT on decision-making (Homberg, 2012). In humans, Macoveanu et al. (2013) reported that administration of the 5-HT_2A_ antagonist ketanserin increased risk aversion, and Faulkner et al. (2014) showed a positive correlation in humans between hippocampal 5-HT_1A_ receptor availability and sensitivity to probability on the gambling task used in Rogers et al. (2003). These studies raise the possibility that specific 5-HT receptor subtypes may moderate the effect of ATD on decision-making. However, this is difficult to test directly in humans due to the high cost and invasive nature of molecular imaging techniques such as positron emission tomography.

Studies have shown that dopamine (Illani et al., 2001) and serotonin (Ye et al., 2014) receptor mRNA levels in the blood correspond to levels in the brain. This was exploited by Ersche et al. (2011), who examined peripheral levels of mRNA pertaining to the dopamine receptor 3 (D_3_) and D_4_ genes in order to understand variability in response to dopamine agonist treatment. Variation in peripheral levels of D_3_ mRNA explained over one-quarter of the variation in improvements on a spatial working memory task due to administration of the D_2/3_ receptor agonist pramipexole. Based on this finding, we reasoned that measuring individual differences in levels of 5-HT receptor mRNA in peripheral blood might help explain variation in the effects of ATD.

The current study aimed to test the effects of ATD on decision-making and to examine whether individual differences in peripheral 5-HT system gene mRNA levels moderate such effects. Whole-blood mRNA levels were measured for 9 5-HT-related genes: 5-HT_1B_, 5-HT_1F_, 5-HT_2A_, 5-HT_2B_, 5-HT_3A_, 5-HT_3E_, 5-HT_7_ (serotonin receptors), 5-HTT (the serotonin transporter), and tryptophan hydroxylase 1 (TPH1). Both standard and computational analyses of decision-making behavior were utilized. Based on previous results examining decision-making under ATD, we predicted that participants would display decreased ability to utilize information pertaining to rewards following ATD; we also predicted that such changes would be related to peripheral 5-HT system mRNA levels.

## Materials and Methods

### Participants

Thirty participants were recruited via online advertisements. All participants gave written informed consent and the University College London (UCL) Research Ethics committee approved the study. Exclusion criteria (assessed by the Mini International Neuropsychiatric Inventory: Sheehan et al., 1998) included past/present major depressive disorder, bipolar disorder, psychosis, anxiety disorders, psychotropic medication usage, substance/alcohol dependence or recent (<6 months) abuse, or any neurological disorder. Participants were medically healthy and none reported taking regular medication for any illness. Both men and women were admitted to the study. However, (female) participants’ menstrual cycle stage was not recorded.

Following exclusions (detailed in the supplementary Online Materials), data from 22 participants were analyzed to assess the biochemical effect of the ATD procedure, data from 26 participants were analyzed to assess ATD effects on decision-making, and data from 21 participants were analyzed to assess relationships between peripheral 5-HT system mRNA levels and ATD-induced effects on decision-making.

### Procedure

Testing took place at the Wellcome Trust Centre for Neuroimaging, UCL. Participants completed 2 testing sessions (ATD and sham) at least 1 week apart and fasted for 8 hours prior to the start of each testing session. At the start of each study day (T0), blood samples were obtained to determine baseline plasma amino acid levels and 5-HT system peripheral mRNA levels. Participants then completed the State Trait Anxiety Inventory (STAI; Speilberger et al., 1987) and ingested an amino acid drink either selectively lacking (TRP-) or containing (sham depletion, TRP+) tryptophan, in a within-subjects, double-blind, sham-controlled design. The order of drink administration was counterbalanced across participants. Participants then rested (and continued to fast) at the testing site for the next 5 hours. Participants then completed the STAI, and blood samples were obtained once more at the end of this 5-hour period (T5) to determine plasma amino acid levels post-treatment before the gambling task was performed (as part of a larger cognitive battery; data for other tasks will be reported elsewhere). Participants were then provided with a tryptophan-rich meal before leaving the laboratory.

The concentrations of each amino acid in the drink were based upon those reported in Young et al. (1985) and are reported in the supplementary Online Materials, together with the methods used for plasma amino acid analysis.

### Gambling Task

Participants completed 80 trials, during each of which they had to make a choice between 2 gambles. Each gamble was represented as a bar, the height of which indicated the probability of winning or losing a number of points. The magnitudes of potential gains and losses were displayed in green at the top and in red at the bottom of each bar, respectively (supplementary Figure 1). One of the gambles, called the control gamble, was the same on every trial. It involved a lower-stakes 50% chance of winning or losing 10 points. The other, experimental, gamble involved either a 75% or 25% chance of winning 80 or 20 points and the inverse chance of losing 80 or 20 points. There were thus 8 trial types in total, which are described in supplementary Table 1. Two additional trial types, “gains only” and “losses only,” were also administered, but data from these were not included in the analyses.

There were 3 main basic (i.e., noncomputational) outcomes variables from this task, namely the proportion of experimental gambles chosen over the control gamble as a function of: (1) the probability of winning (high vs low probability of winning); (2) the magnitude of potential win (high vs low magnitude); and (3) the magnitude of potential loss (high vs low magnitude) (supplementary Figure 1), which we term “sensitivity” to probability, win, and loss after Rogers et al. (2003). Each of these 3 measures was calculated by taking the difference between the proportion of experimental gamble choices when each of these factors was high compared with when it was low. For clarity in presenting the results, the negative of loss sensitivity is depicted.

### Questionnaire

The STAI is a 40-item, self-rating anxiety measure; the first 20 items identify state anxiety, and the second 20 identify trait anxiety. Participants must give a score of 1 (“do not agree at all”), 2 (“agree somewhat”), 3 (“agree moderately”), or 4 (“very much agree”). Scores are then summed to give their state and trait anxiety scores.

### mRNA Extraction and Analysis

Blood samples (4mL) were drawn into PAXgene tubes (Qiagen) to maximize the stability of the mRNA and avoid potential degradation. Levels of plasma mRNA were examined for 5-HT_1A_, 5-HT_1B_, 5-HT_1D_, 5-HT_1E_, 5-HT_1F_, 5-HT_2A_, 5-HT_2B_, 5-HT_2C_, 5-HT_3A_, 5-HT_3B_, 5-HT_3C_, 5-HT_3D_, 5-HT_3E_, 5-HT_4_, 5-HT_5A_, 5-HT_6_, 5-HT_7_, 5-HTT, TPH1, and TPH2. However, mRNA levels were detectable for all subjects for only 5-HT_1B_, 5-HT_1F_, 5-HT_2A_, 5-HT_2B_, 5-HT_3A_, 5-HT_3E_, 5-HT_7_, 5-HTT, and TPH1, and as such only these transcripts were included in the correlational analyses. Details of sample processing and mRNA measurement are provided in the SOM.

### Statistical Analysis

Data were analyzed using IBM SPSS Statistics (http://www-01.ibm.com/software/analytics/spss/products/statistics). An arcsine (square root) transform was applied to probability data prior to analysis. The order of treatment administration (i.e., TRP- in week 1 or week 2) was added as a between-subjects factor for all analyses.

Plasma amino acid data were analyzed using a 2x2 ANOVA, with treatment constituting 2 levels (ATD and sham) and time constituting 2 levels (T0 and T5). Decision-making data were analyzed using a 2x2x2x2 ANOVA, with treatment constituting 2 levels, and each of probability, win, and loss constituting 2 levels (high and low); based on our a priori hypotheses, we focused on the effects of probability, win, and loss and their interactions with treatment. Mood data from the STAI were analyzed using a 2x2 ANOVA, with treatment constituting 2 levels and time constituting 2 levels (T0 and T5). Significant interactions were interrogated by constructing the simple main or interaction effects. A significance threshold of alpha=0.05 (2-tailed) was adopted for all analyses, while .05<*P*<.10 was considered a trend towards significance. Relationships between peripheral 5-HT system mRNA levels and ATD-induced changes in decision-making behavior were analyzed using Pearson’s correlation coefficients. Bonferroni correction (BC; for 9 transcripts*4 decision-making measures=36 tests) was applied to the correlational analyses.

Finally, exploratory analyses were conducted to examine whether the effects of treatment, and correlations between treatment-induced changes in task performance and serotonin gene transcription, were moderated by gender. To examine the former, a 2x2x2x2 ANOVA was performed, with treatment (ATD and sham) and each of probability, win, and loss (high and low) as within-subjects factors and gender added as a between-subjects factor. The effect of gender on relationships between peripheral 5-HT system mRNA levels and ATD-induced changes in decision-making behavior was analyzed using general linear models, with the treatment-induced change in sensitivity to probability, win sensitivity, loss sensitivity, and the proportion of experimental gambles acting as dependent variables in separate ANOVAs, peripheral 5-HT system mRNA levels added as continuous covariates, and gender added as a between-subjects factor. The interaction between gender and mRNA levels was included to test for modulatory effects.

### Power Analysis

For the effects of ATD upon decision-making, with data from 26 participants in a within-subjects design, this study had approximately 95% power to detect an effect size of d~0.75 (large, Cohen et al., 1988), comparable with that observed in the study of Rogers et al. (2003) (using a between-subjects design). For correlations between peripheral 5-HT system mRNA levels and the effect of ATD on decision-making, with data from 21 participants, this study had 80% power to detect a correlation of r~0.55, comparable with the correlation between peripheral D_3_ mRNA levels and pramipexole-induced improvement in working memory observed by Ersche et al. (2011).

### Computational Analysis

To explore behavior on the decision-making task in more detail, we performed a hierarchical Bayesian mixed-effects analysis of participants’ data (Daw, 2009), fitting a set of 5 increasingly complex, parametrized logistic regression models (supplementary Table 2). The models included different components involving information about probability, reward, expected values, and other factors.

All statistical analyses were based on permutation tests with empirical null distributions created by randomizing TRP status across testing sessions. Posterior distributions over the parameters for each model for each participant and condition were estimated using a Hamiltonian Monte Carlo sampler, implemented in PyStan (http://mc-stan.org/pystan.html). We additionally generated synthetic data from our winning model to assess the fidelity with which it could recapitulate the statistical characteristics of the original behavior. Further details are provided in the supplementary Online Material.

## Results

### Plasma Amino Acid Concentrations

Total tryptophan concentration (nmol/mL) in the plasma was lower overall on the TRP- day than the TRP+ day (significant main effect of treatment: F(1,20)=160.712, *P*<.001) and was lower overall at T5 than T0 (significant main effect of time: F(1,20)=9.189, *P*=.007). As expected, there was a significant treatment-by-time interaction (F(1,20)=133.174, *P<*.001): plasma tryptophan concentration increased 126.5% from T0 to T5 on the TRP+ day (mean T0=56.16, SD=12.50, mean T5=127.24, SD=40.42, t(21)=7.994, *P<*.001) and decreased 73.3% from T0 to T5 on the TRP- day (mean T0=60.39, SD=13.97, mean T5=16.12, SD=5.66, t(21)=19.239, *P<*.001). There was no difference in mean tryptophan concentrations between TRP+ and TRP- days at T0 (t[21]=1.343, *P*=.194). There was no effect of drink order on tryptophan levels (F(1,20)=.211, *P*=.651), and no significant interaction with order.

The tryptophan:large neutral amino acid (LNAA) ratio was also lower on the TRP- day than the TRP+ day (significant main effect of treatment: F(1,20)=102.475, *P<*.001) and was lower at T5 than T0 (significant main effect of time: F(1,20)=29.972, *P<*.001). Again, there was an expected treatment-by-time interaction (F(1,20)=73.105, *P<*.001): the plasma ratio increased 17.2% from T0 to T5 following TRP+, which showed a trend towards significance (mean T0=0.156, SD=0.046, mean T5=0.189, SD=.015, t(21)=1.931, *P*=.067), and significantly decreased 85.1% from T0 to T5 following TRP- (mean T0=0.166, SD=0.054, mean T5=0.025, SD=0.012, t(21)=13.860, *P<*.001). Again, there was no difference between the days on the plasma ratio measure at T0 (t[21]=0.937, *P*=.359), no effect of drink order on the ratio measure (F(1,20)=.541, *P*=.470), and no significant interaction with order.

### Decision-Making Task: Proportionate Choice

Choice data are displayed in Table 1A. Participants chose the experimental gamble significantly more often when the probability of winning was high (F(1,24)=223.812, *P<*.001), when the amount that could be won was high (F(1,24)=34.798, *P<*.001), and when the amount that could be lost was low (F(1,24)=34.364, *P<*.001).

**Table 1.** Proportion (SD) of Choices of the Experimental Gamble (A), and Mean (SD) Deliberation Times (ms; B) as a Function of the Probability of Winning, the Magnitude of Potential Wins, and the Magnitude of Potential Losses under ATD (TRP-) and Sham (TRP+)

	Probability of Winning		Magnitude of Wins		Magnitude of Losses	
A	High	Low	Large	Small	Large	Small
TRP-	0.77 (.18)	0.18 (.19)	0.55 (.12)	0.39 (.12)	0.38 (.16)	0.56 (.10)
TRP+	0.84 (.15)	0.17 (.14)	0.58 (.10)	0.44 (.12)	0.42 (.14)	0.60 (.09)
B	High	Low	Large	Small	Large	Small
TRP-	1766 (1015)	2013 (1055)	1902 (1078)	1878 (1008)	1935 (1066)	1845 (1018)
TRP+	1769 (993)	1983 (930)	1882 (1006)	1871 (930)	1970 (993)	1793 (935)

Following TRP- participants displayed a trend towards choosing the experimental gamble less often compared with TRP+ (F(1,24)=3.532, *P*=.072). Examining each sensitivity separately, there was no effect of treatment upon sensitivity to probability (F(1,24)=0.668, *P*=.422), wins (F(1,24)=0.210, *P*=.651), or losses (F(1,24)=0.143, *P*=.708).

There was no effect of drink order on participants’ overall choices (F(1,24)=0.007, *P*=.936). There was a significant drink order-by-treatment-by-probability interaction (F(1,24)=6.445, *P*=.018), indicating a practice effect by which sensitivity to probability was higher on the second testing session. No interactions between order, treatment, and either wins or losses approached significance (both *P*>.169).

### Deliberation Times

Deliberation time data are displayed in Table 1B. Participants were significantly quicker to respond when the probability of winning was high (F(1,24)=7.437, *P*=.012) and when the magnitude of potential losses was low (F(1,24)=6.887, *P*=.015), but the effect of the magnitude of potential wins on reaction time was nonsignificant (F(1,24)=.017, *P*=.898).

There was no main effect of treatment on participants’ deliberation times (F(1,24)=1.102, *P*=.304). Treatment did not significantly influence participants’ deliberation times as a function of probability, potential wins, or potential losses (all interactions: *P*>.321). There was a significant drink order-by-treatment interaction on participants’ deliberation times (F(1,24)=14.625, *P*=.042), indicating a practice effect by which responses were faster on the second testing session. There was also a significant drink order-by-treatment-by-loss interaction (F(1,24)=5.446, *P*=.028). The increase in deliberation times on high potential loss trials was attenuated on the second testing session, again indicating a practice effect. No interactions between order, treatment, and either probability or wins approached significance (both *P*>.292).

### Effect of Acute Tryptophan Depletion on Anxiety

There was no significant main effect of treatment upon participants’ state anxiety scores (state STAI: F(1,24)=.121, *P*=.732). There was also no main effect of time on state STAI (F(1,24)=2.211, *P*=.153). However, there was a significant treatment*time interaction (F(1,24)=6.651, *P*=.019), with posthoc paired *t* tests revealing that while scores did not differ significantly between T0 and T5 on the TRP+ day (mean (SD) T0 TRP+=10.71 (9.13), T5 TRP+=10.33 (8.32)), participants’ state STAI scores increased significantly from T0 to T5 on the TRP- day (T0 TRP-=9.86 (5.94), T5 TRP-=12.00 (6.72), t(25)=2.918, *P*=.009).

### Associations between Peripheral Serotonin mRNA Expression and ATD Effects on Decision-Making

**Figure 1.**
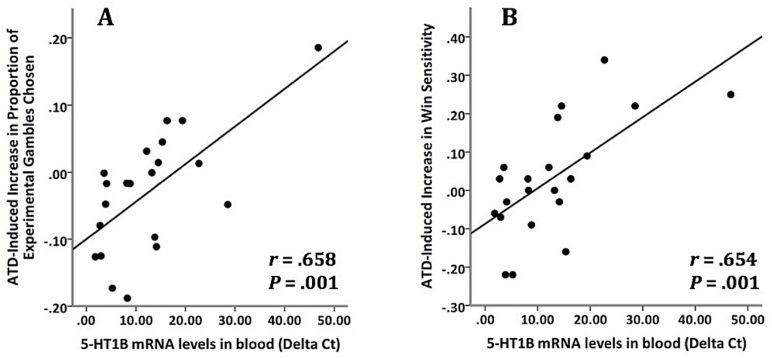
Relationships between baseline peripheral serotonin (5-HT)_1B_ mRNA levels and the treatment-induced increase (acute tryptophan depletion [ATD] minus sham) in the overall proportion of experimental gambles chosen (A) and the ATD-induced increase in sensitivity to wins (B).

There was a significant positive correlation between baseline peripheral 5-HT_1B_ mRNA levels and the ATD-induced increase (TRP- minus TRP+) in the overall proportion of experimental gambles chosen (r=.658, nominal *P*=.001, Bonferroni-corrected P_BC_=.041), with participants with higher baseline 5-HT_1B_ transcription levels displaying the greatest increase (Figure 1A). There was also a significant positive correlation between baseline 5-HT_1B_ transcription levels and the ATD-induced increase in win sensitivity (TRP- minus TRP+: r=.654, nominal *P*=.001, P_BC_=.045), with participants with higher baseline 5-HT_1B_ transcription levels displaying the greatest increase (Figure 1B). There was no other nominally significant correlation between peripheral 5-HT system mRNA levels and ATD-induced changes in decision-making.

### Associations between Peripheral Serotonin mRNA Expression and ATD Effects on Anxiety

There was no significant correlation between peripheral transcription levels of serotonin receptor mRNA and the ATD-induced increase (TRP- T5 minus TRP- T0 scores) in state STAI scores (all *P*>.182).

### Effect of Sex on Responses to ATD

We conducted exploratory analyses examining the effect of sex on treatment-induced changes in task performance. Results of the repeated-measures ANOVA revealed no significant interactions between gender and treatment (F(1,24)=1.848, *P*=.187), between gender, treatment, and sensitivity to probability (F(1,24)=.373, *P*=.547), between gender, treatment, and sensitivity to wins (F(1,24)=.083, *P*=.776), or between gender, treatment, and sensitivity to losses (F(1,24)=.028, *P*=.868).

We conducted exploratory analyses examining the effect of gender on the relationship between treatment-induced differences in both win sensitivity and the overall proportion of experimental gambles chosen with baseline serotonin receptor transcription levels. Regarding win sensitivity, there was an expected significant main effect of baseline 5HT_1B_ transcription levels (*F*(1,17)=11.697, *P*=.003) but no significant main effect of gender (*F*(1,17)=1.334, *P*=.264) and no significant interaction between baseline 5HT_1B_ transcription levels and gender (*F*(1,17)=.201, *P*=.660). Regarding the overall proportion of experimental gambles chosen, there was an expected significant main effect of baseline 5HT_1B_ transcription levels (*F*(1,17)=8.296, *P*=.010), but no significant main effect of gender (*F*(1,17)=2.504, *P*=.132) and no significant interaction between baseline 5HT_1B_ transcription levels and gender (*F*(1,17)=.2.575, *P*=.127). All models examining the effect of gender on the relationship between transcription levels of the remaining serotonin genes revealed no main effects of transcription levels or gender and no significant interactions between transcription levels and gender.

### Computational Analysis

We considered a family of models involving different potential influences of probability, reward, and expected value and included a trial-type independent choice bias parameter (sometimes called a lapse rate) to account for rare, unlikely decisions.

**Figure 2.**
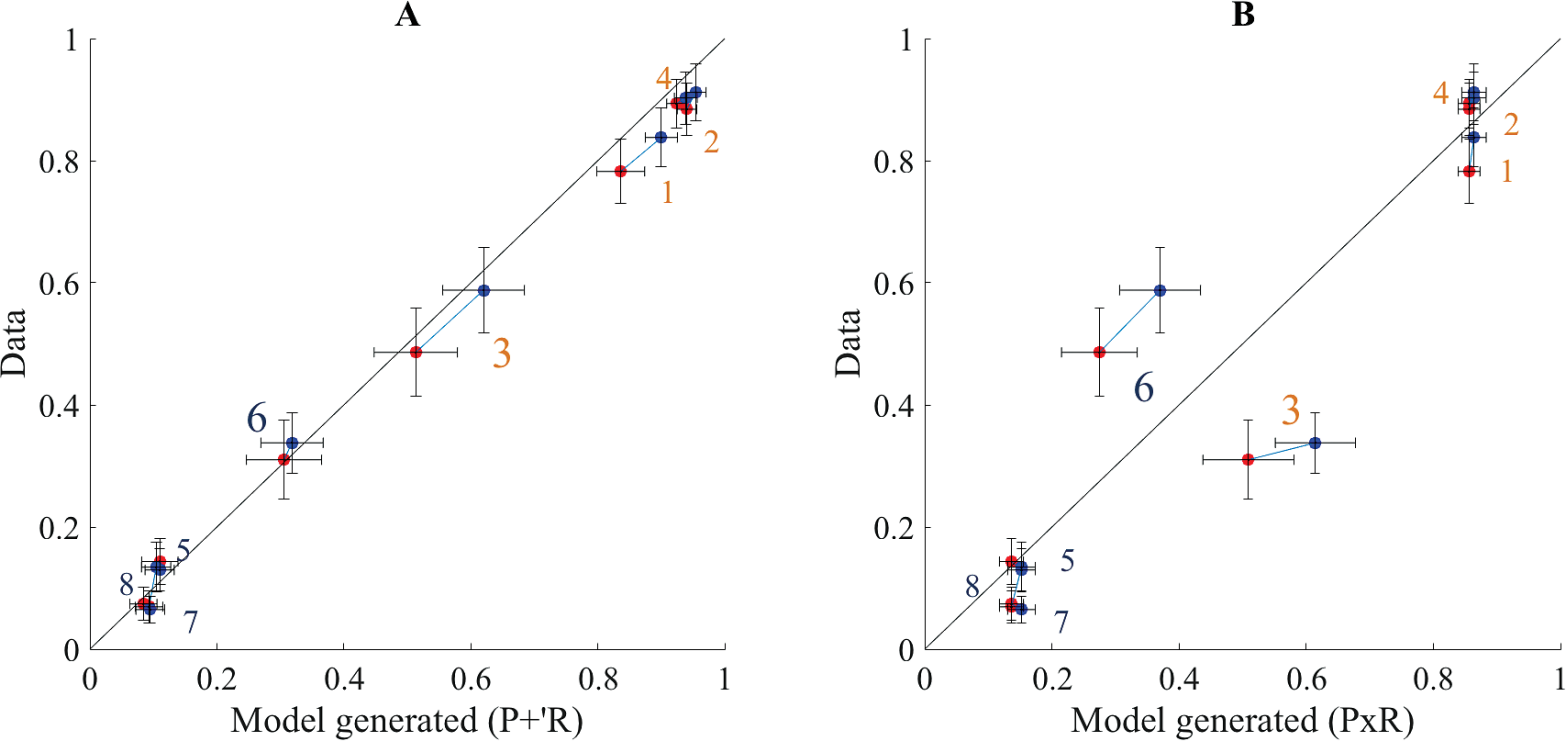
Calibration plots for models P+’R (A) and PxR (B). Red dots: tryptophan (TRP)- condition; blue dots: TRP+ condition. The x-axis represents model predictions (means of synthetic data derived using the best-fitting parameters); the y-axis the frequencies as computed from the raw data. Numbers indicate trial types (see supplementary Materials for details) with 3 and 6 displayed in larger fonts to emphasize that they elicit more variability in responses; different colors of numbers indicate high (orange) and low (blue) probabilities of winning. Error bars indicate inter-subject standard errors as extracted from the raw data (vertical) and subject-level synthetic data derived from model fits (horizontal).

We first identified the most parsimonious model to parameterise performance on the decision-making task, independent of depletion condition. Widely Applicable Information Criterion (WAIC) scores and log predictive densities (LPD) for the 5 models are displayed in supplementary Table 3. Model P+R (in which probability and reward exert additive influences on choice; calibration plot in Figure 2A) is the winning model (lowest WAIC score). By contrast, model PxR, which parametrizes conventional utility theory (i.e., it assumes that subjects multiply probabilities by outcomes to calculate the expected values for each option) performs poorly, reflected in its relatively high WAIC score and more negative LPD (denoting high deviation between model predictions and participants’ behavior) (Figure 2B). The extra complexity of model P+R+(PxR), which allows for both additive and multiplicative influences of probability and reward, is not justified by the improved fit (less negative LPD), although the WAIC score is very close to that of model P+R.

After having recognized the most accurate model (P+R), we slightly refined the prior distributions over the sensitivity parameters and included a condition-independent lapse rate as described in the supplementary Materials (we denote this model P+’R). Total variation in choice frequencies (which was generally low, other than for 2 trial types, possibly limiting experimental sensitivity) is discussed in the SOM and depicted in supplementary Figure 3. The parameters associated with models PxR and P+’R are reported in Table 2. The close correspondence across subjects between participants’ actual and modelled sensitivities to probability, wins, and losses (from the winning model P+’R), displayed in supplementary Figure 2, suggests that the model accurately recapitulated the patterns observed in the raw data.

**Table 2.** Sample Means (SDs) for the Parameter Distributions (α) of Models PxR (multiplicative) and P+’R (Additive, the Winning Model) Including Lapse Rate

Model PxR	α_*PW*_	α_*PL*_		Bias	Lapse Rate
TRP-	1.59 (0.36)	1.77 (0.37)		-0.08 (0.67)	0.28 (0.15)
TRP+	1.67 (0.36)	1.66 (0.34)		0.06 (0.68)	0.28 (0.18)
Model P+’R	α_*P*_	α_*W*_	α_*L*_		
TRP-	0.22 (0.10)	0.067 (0.008)	0.085 (0.022)	0.44 (0.62)	0.14 (0.16)
TRP+	0.23 (0.10)	0.068 (0.007)	0.079 (0.016)	0.73 (0.68)	0.14 (0.17)

Abbreviations: L, loss; PL, probability*loss; PW, probability*win; W, win.

Descriptive statistics were computed using permuted samples from the posterior distributions of population parameters. *Bias* indicates the tendency to choose the experimental (when positive) or control gamble (when negative). The lapse rate column indicates the proportion of trials in which choice was inferred as being independent of trial-type (0, none, 1, all trials).

Consistent with the standard noncomputational analyses reported above, we found no significant effect of ATD on the computationally derived parameters from the winning P+’R model (Table 2; probability parameter: t(25)=0.653 *P*=0.520; win parameter: t(25)=0.494, *P*=0.626; loss parameter: t(25)=1.540, *P*=0.136). We also found no significant effect of ATD on other computationally obtained measures (lapse rate: t(25)=.052, *P*=0.959; trial-type independent bias: t(25)=1.889, *P*=0.071).

### Associations between Peripheral Serotonin mRNA Expression and ATD Effects on Decision-Making Assessed Using Computational Analysis

**Figure 3.**
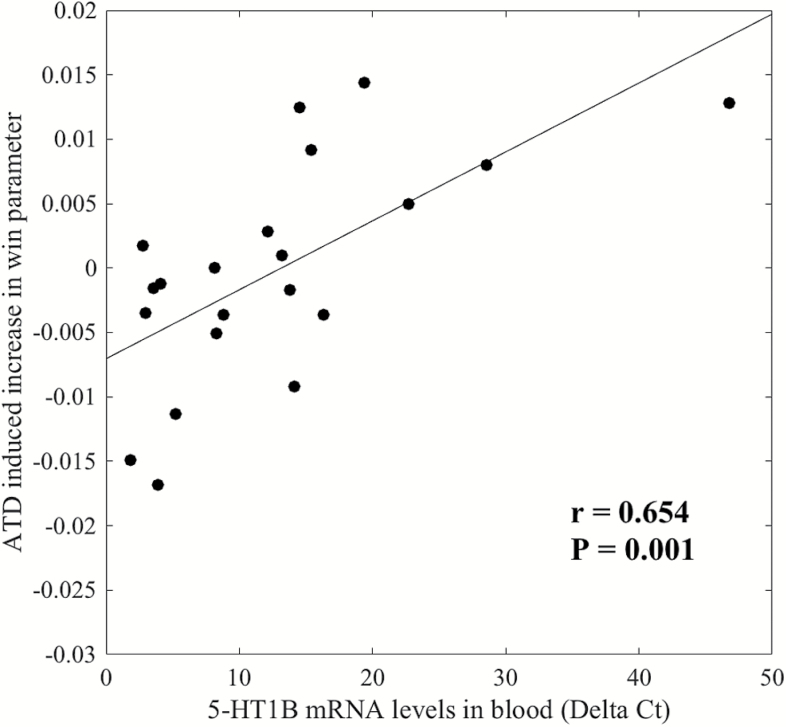
Correlation between peripheral serotonin (5-HT)_1B_ mRNA levels and the acute tryptophan depletion (ATD)-induced increase (tryptophan [TRP]- minus TRP+) in the win parameter (from model P+’R).

Finally, we assessed correlations between the effect of ATD on our fitted parameters (from model P+’R) and the transcripts discussed above. We found a positive correlation between the ATD-induced increase (TRP- minus TRP+) in the win parameter and peripheral 5-HT_1B_ transcription levels (r=0.654, nominal *P*=.001, Figure 3), which survives correction for multiple comparisons (*P*_BC_=.035) and mimics the correlation evident in the standard noncomputational analyses above (Figure 1B).

## Discussion

Several studies have suggested that depleting central serotonin levels, via acute tryptophan depletion, affects reward processing during decision-making (e.g., Rogers et al., 2003; Seymour et al., 2012). However, no previous study has attempted to delineate the roles of specific 5-HT receptors in this effect. We were unable to detect statistically significant effects of ATD on risky decision-making on the gambling task, contrary to the original report (Rogers et al., 2003). However, using an individual differences approach, we identified that higher baseline 5-HT_1B_ mRNA levels in the blood predicted greater ATD-induced increases in both the number of experimental gambles chosen and sensitivity to wins. This pattern was confirmed through detailed computational analysis. Our computational analysis also suggested that, independent of ATD, subjects tend to perform this gambling task by treating information pertaining to probability, wins, and losses additively, not multiplicatively as would be predicted by standard utility theory.

The finding that ATD did not significantly affect participants’ discrimination between the magnitude of expected gains fails to corroborate the results of Rogers et al. (2003). The discrepancy between these results is unlikely to be due to the efficacy of the depletion; participants in the current study experienced, on average, a 73% decrease in plasma tryptophan levels, which is only 10% less than the decrease observed in Rogers et al. (2003) and is higher than the decrease in plasma tryptophan levels observed in other studies that show ATD to affect cognitive performance (e.g., Crockett et al., 2012). This difference is also unlikely to be related to design differences between the 2 studies. We used an identical task, though for many of the trial types we found that variability across participants and conditions (and therefore statistical sensitivity) was very low (supplementary Figure 3). Although we used a within-subjects design, we found little evidence for repetition effects on the task, and with 26 participants we had >95% power to detect a “large” effect size of d~0.75 (Cohen et al., 1988), as reported by the original study.

Another possible reason for the discrepancy with the results of Rogers et al. (2003) could be variation in the sample populations: There was a modest difference in mean age between subject samples, with the average age of the 26 participants included in our behavioral analyses being 29.5 years, while those in Rogers et al. (2003) were on average 23.8 years. However, there is no literature indicating that such a small age difference should account for the reported differences between results. There is some evidence, however, that ATD can have differing, and even directionally opposite effects, depending on trait characteristics that were not measured in the current study, such as aggression (Bjork et al., 2000) and a family history of alcoholism (Crean et al., 2002). Given that the current results show peripheral levels of 5-HT_1B_ mRNA to predict responses to ATD, it would be remiss to discount the influence of other such individual differences.

The finding that peripheral 5-HT_1B_ transcription levels were related to ATD-induced changes, in both the overall frequency of experimental gambles chosen and sensitivity to wins, provides direct evidence that peripheral 5-HT biomarkers can be used to predict responses to ATD. Apart from Ersche et al. (2011), who reported that variation in peripheral D_3_ receptor mRNA levels explained over one-quarter of the variance in pramipexole-induced improvements on a spatial working memory task, attempting to predict inter-individual variation in cognitive responses to treatment by examining peripheral transcriptional biomarkers has not been done. Our results are the first to suggest that such biomarkers can predict response to a treatment that acts directly on the serotonin system. With currently established procedures that measure serotonin markers being both invasive and expensive (e.g., PET), it may be useful to obtain peripheral 5-HT markers when attempting to predict individual responses to clinical treatments that act upon the 5-HT system.

Very few studies have attempted to examine the roles of specific 5-HT receptors in decision-making in humans. For example, Macoveanu et al. (2013) reported that administering the 5-HT_2A_ antagonist ketanserin increases risk aversion, and we (Faulkner et al., 2014) found that hippocampal 5-HT_1A_ receptor availability is related to sensitivity to the probability of winning on the same gambling task adminsitered in the present study. However, while a link between decision-making and the 5HT_1B_ receptor has been observed in mice (Pattij et al., 2003), very few studies have reported a relationship between risky decision-making and this receptor, particularly in humans. Potenza et al. (2011) report that greater 5-HT_1B_ availability within the ventral striatum and putamen, as shown by PET, was related to greater scores on the South Oaks Gambling Screen in problem gamblers. In addition, Curley et al. (2013) showed that, compared with placebo, administration of the potent 5-HT_1B_ agonist trifluoromethlyphenylpiperazine increased BOLD activation within the putamen when anticipating a reward during performance on a gambling task.

While these studies indicate a potential role for striatal 5-HT_1B_ receptors in risky decision-making, the function of this receptor is thought to differ depending upon its location. For example, 5-HT_1B_ receptors within the striatum are thought to inhibit 5-HT release (Pytliak et al., 2011), whereas such receptors within the frontal cortex are thought to inhibit dopamine release (Sarhan et al., 1999). Decreases in both 5-HT and dopamine are thought to mediate risky decision-making and decrease the processing of information pertaining to rewards (see Rogers et al., 2011 for a review). Given that activity within the striatum and orbitofrontal cortex has often been associated with risky decision-making (e.g., Peters and Büchel, 2009), future studies could examine whether the observed correlation between 5-HT_1B_ mRNA levels and ATD-induced changes in risky decision-making is mediated by activation changes within this circuit and by activity within the 5-HT or dopamine system.

This study has several limitations. First, while Ye et al. (2014) suggest that 5-HT receptor mRNA levels in blood are related to levels in the brain (midbrain and diencephalon) in mice, direct evidence for this in humans is lacking. While there are clear similarities between peripheral and brain levels of mRNA, variation between these 2 tissue types does exist (Sullivan et al., 2006, Le-Niculescu et al., 2009). However, Cattaneo et al. (2010) argue that treatment-induced increases in peripheral mRNA levels are a valid proxy for treatment-induced increases in mRNA levels in the brain by reporting that selective serotonin reuptake inhibitor-induced increases in peripheral neurotrophic factor mRNA in blood are comparable with such increases previously reported in brain (see Autry and Monteggia, 2012, for a review). However, the lack of direct evidence that 5-HT receptor mRNA levels in blood are related to those in the brain in humans means that work aimed at validating this approach could be useful. Second, while we attempted to examine transcription levels for 20 5-HT genes, due to low levels in the blood we were able to obtain reliable measurements for only 9. The literature is beginning to shed light on the roles of 5-HT receptor subtypes other than 5-HT_1B_ in decision-making, such as the 5-HT_1A_ and 5-HT_2A_ (Macoveanu et al., 2013; Faulkner et al., 2014), and testing for the existence of relationships between risky decision-making and peripheral levels of mRNA for other 5-HT receptors could have proved useful. Third, the sample size used to assess the relationship between our peripheral markers and treatment-induced alteration in task performance was rather low. While the effect size was strong and we corrected for multiple comparisons to increase confidence in our findings, small sample sizes can sometimes overestimate effect sizes and replication may therefore be useful.

In summary, ATD did not significantly influence participants’ sensitivity to probability, wins, or losses, although there was a trend towards a lower general tendency to choose experimental gambles following depletion. As such, our adequately powered study failed to replicate the results of Rogers et al. (2003), in which depletion decreased participants’ ability to discriminate between the magnitudes of expected gains. However, following ATD, participants with higher peripheral 5-HT_1B_ transcription levels increased their overall choice of experimental gambles (relative to sham depletion) and used information pertaining to possible wins more when making their choices, a result that was confirmed through detailed computational analyses. These results suggest that the 5-HT_1B_ receptor may modulate the effects of ATD on risky decision-making and indicate the utility of using peripheral 5-HT system mRNA levels to predict the efficacy of treatments that perturb this neurotransmitter system.

## Supplementary Material

Supplementary_Material
