# Supplementary material for: Peripheral Serotonin 1B Receptor Transcription Predicts the Effect of Acute Tryptophan Depletion on Risky Decision-Making
Source: Int J Neuropsychopharmacol. 2016 Sep 16;20(1):58–66. doi: 10.1093/ijnp/pyw075 (PMC5480594; doi:10.1093/ijnp/pyw075)
Supplement: Supplementary_Material [file pyw075_suppl_Supplementary_Material.docx]

**Supplementary Material**

**Methods**

**Participant Exclusions**

Of the 30 participants admitted to the study, 3 were excluded from any analyses (2 failed to return for the second testing session, 1 vomited due to ingestion of the amino acid mixture and did not complete behavioral testing). Of the remaining 27 participants, data from only 22 (14 males, mean [SD] age = 31.24 [8.12] years, range = 22-51 years) were included in the analysis of the biochemical effect of ATD (plasma amino acid analyses), as difficulties in taking blood meant that plasma samples were not available at all time points for 5 participants. For the decision-making task, 1 participant was excluded due to failure to understand task instructions, leaving 26 participants (12 male, mean [SD] age 29.45 [12.08] years, age range 24-51) included in this analysis. Of these, only 21 (12 male, mean [SD] age 30.81 [8.48] years, age range 22-51) could be included in the examination of relationships between peripheral 5-HT system mRNA levels and ATD-induced effects on decision-making, due to mRNA quality being too low for 5 participants.

Two participants (both female) did not ingest the full amino acid mixture on both days (one ingested ~75% and the other ~66%). One did not provide a blood sample at all 4 time points and was thus excluded from the amino acid analyses; however, both completed behavioral testing on both sessions. To ensure that the lower amount of amino acid mixture ingested by these 2 participants did not affect the results on the gambling task, analyses of the behavioral data were repeated excluding these participants’ data, but in no case did this alter the results.

**Decision-Making Task**

| **Trial Type** | **Probability** | **Win** | **Lose** |
| --- | --- | --- | --- |
| 1 | .75 | 80 | 80 |
| 2 | .75 | 80 | 20 |
| 3 | .75 | 20 | 80 |
| 4 | .75 | 20 | 20 |
| 5 | .25 | 80 | 80 |
| 6 | .25 | 80 | 20 |
| 7 | .25 | 20 | 80 |
| 8 | .25 | 20 | 20 |

Probabilities of winning, potential wins, and potential losses for each experimental gamble type are reported.

Each choice was represented as a bar, the height of which indicated the probability (75% or 25%) of winning or losing a number of points. The magnitudes of potential gains and losses were displayed in green at the top and in red at the bottom of each bar, respectively.


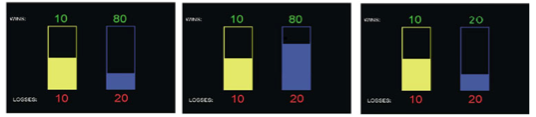


Figure 1. Example trials, showing the control gamble (yellow), consisting of a 50% chance of winning or losing 10 points, and the experimental gamble (blue). Possible wins are in green at the top of the bar, and possible losses are in red at the bottom.

**Amino Acid Concentrations**

TRP+: L-alanine, 4.1 g; L-glycine, 2.4 g; L-histidine, 2.4 g; L-isoleucine, 6 g; L-leucine, 10.1 g; L-lysine, 6.7 g; L-phenylalanine, 4.3 g; L-proline, 9.2 g; L-serine, 5.2 g; L-threonine, 4.9 g, L- tyrosine, 5.2 g; L-valine, 6.7 g, L-arginine 3.7 g, L-cysteine, 2.0 g;, L-methionine, 2.3 g; L- tryptophan, 3 g; total: 78.2 g.

TRP-: L-alanine, 4.1 g; L-glycine, 2.4 g; L-histidine, 2.4 g; L-isoleucine, 6 g; L-leucine, 10.1 g; L-lysine, 6.7 g; L-phenylalanine, 4.3 g; L-proline, 9.2 g; L-serine, 5.2 g; L-threonine, 4.9 g, L-tyrosine, 5.2 g; L-valine, 6.7 g, L-arginine 3.7 g, L-cysteine, 2.0 g; L-methionine, 2.3 g; total: 75.2 g.

The same amounts of amino acids were administered to male and female participants.

**Analysis of Plasma Amino Acid Levels**

Plasma was separated by centrifugation for 10 minutes at 3000 rpm and then stored at -20°C. The total concentrations of large neutral plasma amino acids (tyrosine, valine, phenylalanine, isoleucine, leucine, and tryptophan) were measured by high-pressure liquid chromatography with fluorescence end-point detection and precolumn sample derivatization adapted from the methods of Furst et al. (1990). The limit of detection used was 5 nmol/mL using a 10-µL volume sample. The inter-assay and intra-assay coefficients of variation were <15% and <10%, respectively.

**mRNA Measurement and Sample Processing**

mRNA was isolated using the PAXgene Blood RNA Extraction kit IVD (Qiagen: http://www.preanalytix.com/products/blood/RNA/paxgene-blood-rna-kit-762164), and mRNA quantity and quality was determined by Nanodrop. Samples were analyzed using Fluidigm 96 Dynamic arrays (Fluidigm cat. no. BMK-M-96.96), with assays performed in triplicate. Total RNA (100 ng in 20 µL) was reverse-transcribed into first-strand cDNA using the High Capacity cDNA Reverse Transcription Kit (ABI, PN4368813), and cDNA samples were amplified according to the Fluidigm Specific Target Amplification Quick Reference Manual (http://www.mscience.com.au/upload/pages/fluidigmtech/fluidigm-real-timepcr-user-guide-151112.pdf). The PCR cycling parameters were 50°C 2 minutes, 95°C 10 minutes, and 40 cycles of 95°C 15 seconds and 60°C 1 minute, and data were collected using the Fluidigm Real-Time PCR analysis Software v3.0.2. Relative: 5-HT_1B_, 5-HT_1F_, 5-HT_2A_, 5-HT_2B_, 5- HT_3A_, 5-HT_3E_, 5-H_T7_, 5-HTT, and TPH1 mRNA levels were calculated by calculating Ct (target gene)-housekeeper Ct value (averaged across GAPDH, GUSB, B2M, RPLPO, and TBP) to generate ΔCt values. The average of the 5 housekeeping genes was used for normalization to reduce noise. The relative expression level was calculated using the 2-ΔΔCt method (Livak and Schmittgen, 2001).

Blood samples were collected prior to both testing sessions for all 21 participants included in the mRNA analyses. However, of these 42 samples, 9 had low mRNA quality and were excluded from the analyses (5 from week 1, 4 from week 2), meaning that mRNA levels for 9 participants were calculated from one sample only. Levels of mRNA from the remaining 12 participants were calculated as the average of the 2 weeks.

**Computational Analysis**

We built a family of models to account for subjects’ choices and fit them using pyStan (Stan Development Team. 2016; http://mc-stan.org), a fully Bayesian Markov chain Monte Carlo sampler. The models parameterized the selection of the gamble according to sensitivities to probability (P), reward (R), and the product of probability and reward that underlies the true expected value (PxR). The equations underlying each model are shown in Table 2, where logit(p) is the uncorrected probability of choosing the gamble (the correction according to a lapse parameter is described below). Here, $\alpha_{P}$, the probability parameter, is multiplied by 0.5 when the probability of winning is high (.75) and by -0.5 when it is low (.25). In all models, except for the pure probability one (model P), we multiplied $\alpha_{P}$ by 60 to ensure that this would lie in the same range as the winning and losing parameters. $\alpha_{W}$and $\alpha_{L}$are the winning and losing parameters. $\alpha_{PW}$and $\alpha_{PL}$are the expected wins and expected losses parameters, as they multiply wins and losses weighted according to the probability of each occurring.

**Table 2. The Five Computational Models Tested**

| **Model** |  |
| --- | --- |
| P | $logit\left( p \right)= \alpha_{p}(-1+2p_{w})$ |
| PxR | $logit\left( p \right)= \alpha_{PW}\left( p_{w}\cdot w \right)- \alpha_{PL}\left( p_{l}\cdot l \right)$ |
| P+R | $logit \left( p \right)= 60 \cdot\alpha_{p}\left( -1+2p_{w} \right)+ \alpha_{W}\cdot w- \alpha_{L}\cdot l$ |
| P+(PxR) | $logit \left( p \right)= 60 \cdot\alpha_{p}\left( -1+2p_{w} \right)+ \alpha_{PW}\left( p_{W}\cdot w \right)- \alpha_{PL}(p_{l}\cdot l)$ |
| P+R+(PxR) | $logit \left( p \right)= 60 \cdot\alpha_{p}\left( -1+2p_{w} \right)+ \alpha_{PW}\left( p_{W}\cdot w \right)- \alpha_{PL}\left( p_{l}\cdot l \right)+ \alpha_{W}\cdot w- \alpha_{L}\cdot l$ |

$p$ is the probability of choosing the experimental gamble. $p_{w},p_{l}$ and $w,l$ are the probabilities and numbers of points for winning and losing respectively; $p_{w}\cdot w,p_{l}\cdot l$ are the expected wins and losses. The models are labelled by the dependencies they include, on main effects or combinations of probability (P), reward (R), and average expected reward (PxR). Model P+’R follows the same equation as model P+R.

A conventional finding in fitting such models is that subjects are rarely completely deterministic, even for choices that are overwhelmingly biased one way or the other. The standard way to capture this is via a trembling hand, or lapse process, a trial-type independent random Bernoulli draw as to whether the choice is determined by the logit(p) of Table 2 or a bias towards choosing the experimental or control gamble. We therefore tested whether this factor improved the fit of the model.

We first considered factorial Gaussian population distributions for the sensitivity parameters across participants, with flat priors over the means and variances of these distributions. We used a beta distribution to characterise the lapse rate distribution at the population level, and a Gaussian distribution to characterise the bias in the lapse process. We treated TRP+ and TRP- conditions separately.

We compared the models based on the WAIC with lower scores denoting a better model (Watanabe, 2010). WAIC can be thought of as an approximation to cross validation for large data sets. This score is calculated by estimating the model’s LPD, which quantifies the expected likelihood of the data under the sampled posterior distributions and then subtracting the number of effective parameters ($n_{eff}$). The resulting quantity is then multiplied by -2, thus becoming a deviance measure. In mathematical terms:

$$WAIC=-2(LPD-n_{eff})$$

Table 3 shows the WAIC scores for the various models.

Posthoc analyses of the parameters of the winning model suggested that there was a notable difference between the magnitudes of the sensitivities to probability vs win and loss. We therefore tested a slight refinement (P+’R) in which win and loss sensitivities were sampled from an uncorrelated bivariate normal distribution and probability sensitivity from an independent gamma distribution. This had a very slightly better WAIC score than model P+R, as also seen in Table 3.

**,**

| **Model** | **WAIC** | **LPD** | **WAIC(+lapse)** | **LPD(+lapse)** |
| --- | --- | --- | --- | --- |
| P | 2902 | -1410 | 2747 | -1297 |
| PxR | 2805 | -1305 | 2770 | -1199 |
| P+R | 2159 | -956 | 2127 | -938 |
| P+’R | 2161 | -970 | 2125* | -937* |
| P+(PxR) | 2288 | -1011 | 2284 | -1027 |
| P+R+(PxR) | 2164 | -946 | 2168 | -948 |

The mean lapse rate across the population, as inferred from model P+’R , was 14%. We report the scores for all models, with and without the inclusion of a lapse rate parameter. The starred WAIC score and LPD denote the values corresponding to P+’R, the best performing model.

Figure 2 shows that the posterior mean sensitivities that emerge from model fitting are closely related to those found directly from the raw data.

**
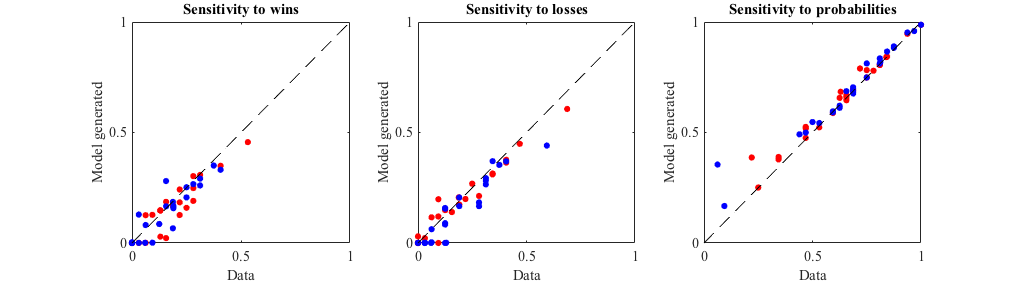
**

Figure 2. Close agreement between sensitivities to potential wins, losses, and probability as computed from the raw data (x-axis), and equivalent sensitivities generated using synthetic data from our best performing model, P+’R (y-axis) (dashed line indicates perfect agreement). The P+’R model-generated sensitivities agree with those obtained from the raw data. In all plots, red dots: TRP- condition, blue dots: TRP+.

As shown in Figure 2 of the manuscript, 6 of the 8 trial types (1, 2, 4, 5, 7, and 8) provide little information about the effects of ATD because of ceiling and floor effects. Figure 3 (below) shows this in a different way. Here, the probability of choosing an experimental gamble was divided into 6 equally sized bins, with bin 1 denoting probability = 0-0.16 and bin 6 = probability 0.84-1. The plots in Figure 3 display the numbers of subjects who chose the experimental gamble with a probability belonging to each probability bin in the TRP- condition (x-axis) and in the TRP+ condition (y-axis). Subjects whose choice behavior is unaffected by the TRP status will contribute to one of the bins on the top-left-to-bottom-right diagonal. Note that light yellow depicts more subjects, dark blue depicts fewer. Trial types 3 and 6 (see Table 1 above) are the only ones that exhibit a high degree of entropy; these are the ones for which there is relevant competition between the potential losses and gains. Unfortunately, it was not possible to fit the parameters of our models solely to these trial types, because the orthogonality of the design is violated, and the posterior distribution for the generating parameters becomes severely unconstrained.


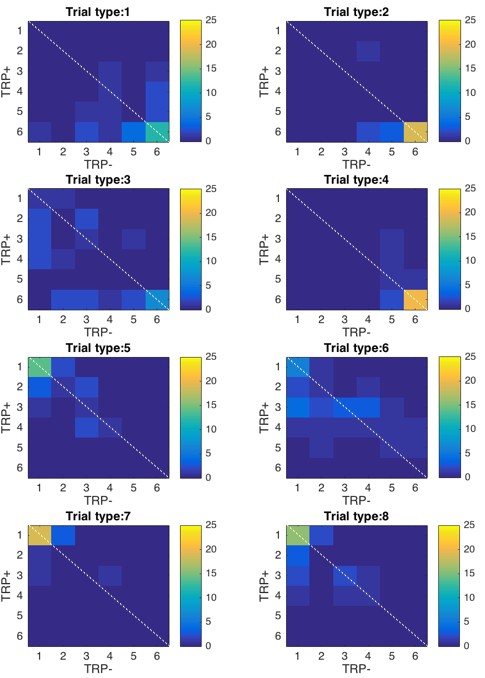


Figure 3. Frequency of experimental gamble choice in the TRP- and TRP+ conditions is divided into equally sized bins, with bin 1 denoting frequencies in [0, 0.16] and bin 6 frequencies in [0.84, 1]. Frequencies are obtained from the raw data. Bin colors indicate the counts of subjects who chose the experimental gamble with a certain frequency under TRP+ (indicated by the bin number on the y-axis) and under TRP- (x-axis). Brighter yellow denotes more participants, while darker blue denotes fewer. A dashed white line from the top left to the bottom right corner of each plot indicates the count of subjects that were unaffected by the TRP status.
